# Premenstrual Disorders, Their Severity Patterns, and Predictors Among Female University Students in Western Uganda: A Cross‐Sectional Study

**DOI:** 10.1002/hsr2.71538

**Published:** 2025-11-23

**Authors:** Marc Nzambimana, Emmanuel Okurut, Marie Pascaline Sabine Ishimwe, Carlos Batista Cedeno, Raissa Marie Ingrid Niyubahwe, Albert Odongo, Suleiman Ali Sleyoum, Christopher Kato, Ahmed Kiswezi Kazigo, Josiah J. Mkojera, Theoneste Hakizimana

**Affiliations:** ^1^ Department of Obstetrics and Gynecology Kampala International University Ishaka Uganda; ^2^ Department of Pediatrics and Child Health Kampala International University Ishaka Uganda; ^3^ Department of Psychiatry Kampala International University Ishaka Uganda; ^4^ Department of Surgery Kampala International University Ishaka Uganda

**Keywords:** predictors, premenstrual dysphoric disorder, premenstrual syndrome, PSST, Uganda, university students

## Abstract

**Background:**

Premenstrual disorders (PMDs), including premenstrual syndrome (PMS) and premenstrual dysphoric disorder (PMDD), are common menstrual‐related conditions that significantly impact the quality of life and academic performance of young women. This study aimed to determine their burden and predictors among female university students in Western Uganda.

**Methodology:**

We conducted a cross‐sectional study (December 2024–March 2025) at Kampala International University, Western Campus using the Premenstrual Symptoms Screening Tool (PSST). The PSST comprises symptom items and functional‐impairment items with validated diagnostic cut‐offs for PMDD and for moderate‐to‐severe PMS. We performed bivariate and multivariable logistic regression to identify predictors of PMDs, reporting adjusted odds ratios (aORs) and 95% confidence intervals (CIs).

**Results:**

A total of 453 participants were enrolled. Overall, 75.9% met PSST diagnostic criteria for a PMD. PMS accounted for 46.3% and PMDD for 29.6% of the sample. Among PMS cases, moderate severity predominated (52.8%), followed by mild (36.2%) and severe (11.0%) categories. Independent predictors of PMDs included enrollment in the School of Allied Health Sciences (aOR = 4.25, 95% CI 1.00–18.03, *p* = 0.050), Muslim religion (aOR = 2.79, 95% CI 1.19–6.54, *p* = 0.020), drug use (aOR = 2.44, 95% CI 1.13–5.26, *p* = 0.024), and history of traumatic events (aOR = 2.24, 95% CI 1.07–4.70, *p* = 0.031).

**Conclusions:**

This study demonstrates a substantial burden of PMDs among female university students, with a predominance of moderate symptom severity. These findings support the need for screening and intervention programs within university settings. Targeted psychosocial support, stress‐management strategies, and menstrual health services may mitigate the academic and emotional consequences of PMDs.

**Abbreviations**
ACOG: american college of obstetricians and gynecologists
DRSP: the daily record of severity of problems
FSH: follicle‐stimulating hormone
ICD‐11: international classification of disease 11th revision
LH: luteinizing hormone
PMDD: premenstrual dysphoric disorder
PMDs: premenstrual disorders
PMS: premenstrual syndrome
PMSST: premenstrual symptoms screening tool
PSQ: premenstrual symptoms questionnaire
REC: research and ethics committee

## 1 Introduction

Premenstrual disorders (PMDs) which include premenstrual syndrome (PMS) and its more severe form, premenstrual dysphoric disorder (PMDD) are common yet frequently under‐recognized conditions that disrupt psychological, physical, and social functioning in women of reproductive age [1, 2]. Many menstruating individuals report at least one premenstrual symptom, but screen‐positive PMS and PMDD represent smaller, clinically significant subsets characterized by impairment; PMDD affects roughly 3%–8% and is often debilitating [3]. Uncertainty about the global burden persists because case definitions, cultural framing of symptoms, and study methods vary widely across settings [4].

Global synthesis suggests substantial prevalence, although estimates differ by measurement approach. Figures as high as 80%–96% generally capture any premenstrual symptom rather than syndromic PMS, for which typical estimates are around 30%–40% in reproductive‐age populations [1]. In Uganda, Odongo et al. reported 97.8% (95% CI 95.2–99.0) of female undergraduates endorsing at least one menstrual complaint, pointing to a high local symptom burden [5]. Country‐level PMS estimates span from 7.7% (United Kingdom) to 98.2% (Pakistan), with a pooled prevalence of 63.5% [1]. PMDD prevalence also varies substantially from 3.2% in the UK to 40% in Egypt, with a global average near 11.4% [6]. In sub‐Saharan Africa, evidence remains limited but suggests notable burden (e.g., 85% in Nigeria; 43.8% in Ethiopia) [7, 8]. These discrepancies underscore the need for context‐specific epidemiology using standardized tools.

Etiology appears multifactorial: sensitivity to normal ovarian steroid fluctuations, genetic predisposition, and psychosocial stressors all contribute [9]. Modifiable correlates, tobacco and alcohol use, caffeine intake, suboptimal diet, low physical activity—and higher body mass index have been linked to increased risk or severity; each unit increase in BMI has been associated with about a 3% higher PMS risk [10, 11]. Early‐life adversity (e.g., childhood trauma, abuse, neglect) is a strong predictor of PMDD in adulthood [12, 13]. Sociodemographic and cultural contexts shape how symptoms are expressed and managed; while overall prevalence may be similar across racial and ethnic groups, symptom profiles differ (e.g., cravings/pain vs. mood swings/fluid retention) and burdens often peak in the third decade of life, especially in the late luteal phase [14, 15].

Clinically, PMDs range from mild, self‐limited symptoms to severe, disabling distress. Up to 40% may experience moderate‐to‐severe manifestations, and 5%–8% meet criteria for PMDD with measurable academic, occupational, and social impairment [2, 6]. These effects are especially salient for university students given high cognitive and emotional demands [1]. Non‐pharmacologic coping exercise, stress management, diet change, social support is common but often informal [16]. Pharmacotherapy, particularly selective serotonin reuptake inhibitors for PMDD, is effective, and hormonal options are used in refractory cases [17]. Nonetheless, stigma, limited awareness, and constrained access to care in low‐resource settings perpetuate under‐diagnosis and suboptimal management [4]. To address these gaps, this study estimates the prevalence, characterizes severity patterns, and identifies associated factors of PMDs among university students in Uganda, generating setting‐relevant evidence using standardized PSST screening criteria.

## 2 Materials and Methods

### 2.1 Study Design and Setting

This descriptive cross‐sectional study was conducted from December 2024 to March 2025 among female university students at the Kampala International University Western Campus (KIU‐WC), which is located in Ishaka Town, Bushenyi District, in western Uganda. KIU‐WC, established in November 2004, is a private institution situated along the Mbarara–Kasese Road and spans approximately 70 acres. It offers a broad range of academic programs, with the Faculty of Clinical Medicine and Dentistry housing the Department of Mental Health and Psychiatry, where this study was anchored.

Bushenyi District is located approximately 340 kilometers southwest of Kampala and is bordered by Rubirizi to the north, Buhweju and Sheema to the northeast, Sheema to the east and south, and Mitooma to the southwest. The district covers an estimated land area of 3,949 square kilometers and lies at an altitude ranging from 910 to 2,500 meters above sea level. The university was purposefully selected to represent the female student population in the region, capturing a wide array of sociodemographic and academic backgrounds. Data collection was conducted over a 3‐month period, from December 2024 to March 2025.

### 2.2 Study Population

The study included female undergraduate students aged 18–49 years at Kampala International University Western Campus who had attained menarche, experienced at least two consecutive menstrual cycles, and provided informed consent. Students were excluded if they were using hormonal contraceptives or medications affecting menstruation, or had bipolar disorder, endocrine disorders, or active symptoms of severe mental illness to minimize confounding. Participants with cycle lengths < 21 or > 38 days were retained, as PSST criteria do not require cycle length exclusions. Sensitivity analysis excluding these participants produced similar results.

### 2.3 Sample Size Calculation

The following sample size formula, Daniels WW, 1999 was used.

$$N=\frac{Z^{2}p(1-p)}{e^{2}}$$

where N is the required sample size estimate and Z is the critical value for a normal distribution at the 95% confidence level, 1.96. P *=* estimated prevalence rate of PMDs, which was 51.4% as reported in the Ethiopian study among university students [18]. q = 1 − *p* Therefore, $N=\frac{1.96^{2}*0.514(1-0.514)}{0.05^{2}}=453\;$

Therefore, the minimum required sample size was 453.

### 2.4 Sampling Techniques

A multistage random sampling approach was employed. First, out of the 12 faculties at the Kampala International University Western Campus, six faculties were purposively selected to represent a diverse and representative student population. These included the Faculties of Clinical Medicine and Dentistry, Allied Health Sciences, Pharmacy, Engineering, Education, and Law.

The total number of female undergraduate students in each selected faculty was obtained. Proportional stratified sampling was then applied to determine the number of participants to be recruited from each faculty, which was based on the proportion of female students in each faculty relative to the total across all six. A final sample of 453 female undergraduate students was selected, and distributed as follows: Clinical Medicine and Dentistry (184 students; 40.6%), Allied Health Sciences (166; 36.6%), Pharmacy (32; 7.1%), Engineering (12; 2.6%), Education (48; 10.6%), and Law (11; 2.4%).

### 2.5 Data Collection Procedure

Following written informed consent, data were collected over 2 months by the principal investigator and a trained assistant. Anthropometric and clinical parameters: including height, weight, waist circumference, and blood pressure were measured via standardized tools and procedures. BMI was calculated and classified according to the WHO criteria, whereas blood pressure was interpreted via the AHA guidelines. Waist circumference was measured at the midpoint between the last rib and iliac crest, with ≥ 80 cm indicating increased risk and ≥ 88 cm indicating substantially increased risk for central obesity.

After providing participants with feedback and health advice on their measurements, a pre‐validated structured questionnaire was administered to assess PMS and PMDD symptoms, along with relevant sociodemographic and lifestyle factors. Research staff remained available for clarification. Participants who screened negative received educational counselling, whereas those who screened positive were guided to seek further evaluation, including referral to gynecological care where appropriate.

### 2.6 Assessment Tool and Diagnostic Criteria

PMDs were assessed using the Premenstrual Symptoms Screening Tool (PSST), which consists of 14 symptom items (e.g., irritability, anxiety, mood swings, low energy, food cravings, insomnia, bloating) and four functional‐impairment items. Each item is rated as not at all, mild, moderate, or severe. Diagnostic cut‐offs followed validated PSST scoring rules: PMDD was diagnosed when ≥ 1 of the first four core mood symptoms was severe, ≥ 4 symptoms were moderate/severe, and ≥ 1 functional‐impairment item was severe. Moderate‐to‐severe PMS required ≥ 1 core mood symptom moderate/severe, ≥ 4 total symptoms moderate/severe, and ≥ 1 functional‐impairment item moderate/severe. The tool was translated into Runyankore‐Rukiga, back‐translated, pretested on 10% of the sample, and demonstrated good internal consistency (Cronbach's *α* = 0.83).

### 2.7 Study Variables

The dependent variable was premenstrual disorder (PMD), encompassing both premenstrual syndrome (PMS) and PMDD. Independent variables included sociodemographic characteristics, gynecological history, and medical and psychological factors.

### 2.8 Quality Control

The data collection tools were pretested among postgraduate students at the Kampala International University Western Campus to assess their clarity, relevance, and reliability. The PSST, which is used to identify PMDD, has demonstrated good validity and reliability (Cronbach's alpha = 0.83) in distinguishing it from conditions like major depressive and anxiety disorders. Pre‐validation involved translation, back‐translation, cognitive debriefing, and pilot testing on 10% of participants. Cronbach's *α* = 0.83 confirmed reliability. Research assistants received thorough training on ethical procedures, informed consent, and standardized data collection protocols. They were closely supervised by the principal investigator to ensure adherence to the study guidelines. The completed questionnaires and anthropometric measurements were reviewed daily for accuracy and completeness. Unfamiliar terms were explained uniformly to all participants, and regular debriefing meetings were held to resolve any issues during data collection.

### 2.9 Data Management and Analysis

The data were first entered and cleaned in Microsoft Excel and then exported to Stata version 15 (StataCorp, College Station, TX, USA) for analysis. Continuous variables such as age, height, weight, BMI, blood pressure, and waist circumference were summarized using means and standard deviations. Categorical variables, including sociodemographic characteristics (e.g., religion, nationality, marital status, year of study), and gynecological and psychological factors (e.g., parity, age at menarche, menstrual cycle length and flow), were summarized using frequencies and percentages.

The prevalence of premenstrual disorders (PMS and PMDD) was calculated as the proportion of participants meeting the diagnostic criteria based on the PSST, with results expressed as percentages and illustrated using pie charts. Symptom severity was categorized as none, mild, moderate, or severe based on PSST scoring criteria, and was summarized descriptively. To identify factors associated with PMS and PMDD, a binary outcome variable was generated (1 = presence of PMS/PMDD, 0 = absence). Bivariate analysis was performed using logistic regression to explore associations between independent variables and PMDs. Variables with a *p*‐value ≤ 0.20 or biological plausibility were entered into a multivariate logistic regression model to identify independent predictors. Adjusted odds ratios (aORs) with corresponding 95% confidence intervals (CIs) were reported, and statistical significance was set at *p* ≤ 0.05.

Multicollinearity was checked using variance inflation factors (VIF < 2.0). Model calibration/discrimination were assessed with the Hosmer–Lemeshow test (*p* > 0.05) and classification metrics. All analyses were two‐sided with α = 0.05. We report odds ratios (OR) with 95% CIs alongside *p* values (formatted per journal guidelines). Analyses were performed using Stata version 15.0 (StataCorp, College Station, TX, USA).

### 2.10 Human Ethics and Consent to Participate

This study was approved by KIU‐REC (KIU‐2024‐484) and registered with UNCST. All participants were ≥ 18 years and gave written informed consent. We followed STROBE guidelines for cross‐sectional studies and SAMPL recommendations for statistical reporting. All participants provided written informed consent after being fully informed about the study's purpose, procedures, and rights. Participation was voluntary, and only individuals aged 18 years or older were enrolled, each providing consent personally.

## 3 Results

### 3.1 Basic Characteristics of the Study Participants

This university‐based cross‐sectional study enrolled a total of 453 female undergraduate students. The majority of participants were aged between 20 and 29 years (93.2%), while a small proportion were below 20 years (3.1%) or between 30 and 39 years (3.7%). Most participants identified as Christian (84.3%) and Ugandan nationals (96.9%). The predominant ethnic groups were Muganda (39.7%) and Munyankole (38.9%), with others accounting for 21.4% of the sample. The students were recruited from six faculties, with the highest representation from the School of Allied Health Sciences (40.6%) and the Faculty of Clinical Medicine and Dentistry (36.6%). A considerable proportion were in their second (41.5%) or third (30.6%) year of study. The majority of the respondents were unmarried (91.6%) (Table 1).

**Table 1:** Sociodemographic characteristics of the study participants (*N* = 453).

Variables	Overall frequency, *N* = 453, *n* (%)	PMD	
		No, *N* = 109, *n* (row %)	Yes, *N* = 344, *n* (row %)
Age category (years)			
< 20	14 (3.1)	5 (35.7)	9 (64.3)
20–29	422 (93.2)	95 (22.5)	327 (77.5)
30–39	17 (3.7)	9 (52.9)	8 (47.1)
Religion			
Christian	382 (84.3)	100 (26.2)	282 (73.8)
Muslim	71 (15.7)	9 (12.7)	62 (87.3)
Nationality			
Ugandan	439 (96.9)	107 (24.4)	332 (75.6)
Non‐Ugandan	14 (3.1)	2 (14.3)	12 (85.7)
Tribe			
Munyankole	176 (38.9)	38 (21.6)	138 (78.4)
Muganda	180 (39.7)	51 (28.3)	129 (71.7)
Others	97 (21.4)	20 (20.6)	77 (79.4)
Faculty			
FCMD	166 (36.6)	38 (22.9)	128 (77.1)
SAHS	184 (40.6)	24 (13.0)	160 (87.0)
Pharmacy	32 (7.1)	9 (28.1)	23 (71.9)
Education	48 (10.6)	29 (60.4)	19 (39.6)
Law	11 (2.4)	3 (27.3)	8 (72.7)
Engineering	12 (2.7)	6 (50.0)	6 (50.0)
Year of study			
First	72 (15.9)	31 (43.1)	41 (56.9)
Second	188 (41.5)	40 (21.3)	148 (78.7)
Third	139 (30.6)	30 (21.6)	109 (78.4)
Fourth	27 (6.0)	2 (7.4)	25 (92.6)
Fifth	27 (6.0)	6 (22.2)	21 (77.8)
Marital status			
Married	38 (8.4)	20 (52.6)	18 (47.4)
Not Married	415 (91.6)	89 (21.5)	326 (78.5)

Abbreviations: FCMD, Faculty of Clinical Medicine and Dentistry; PMD, premenstrual disorder; SAHS, School of Allied Health Sciences.

### 3.2 Prevalence of Premenstrual Disorders Among University Students at KIU‐WC

**Figure 1:**
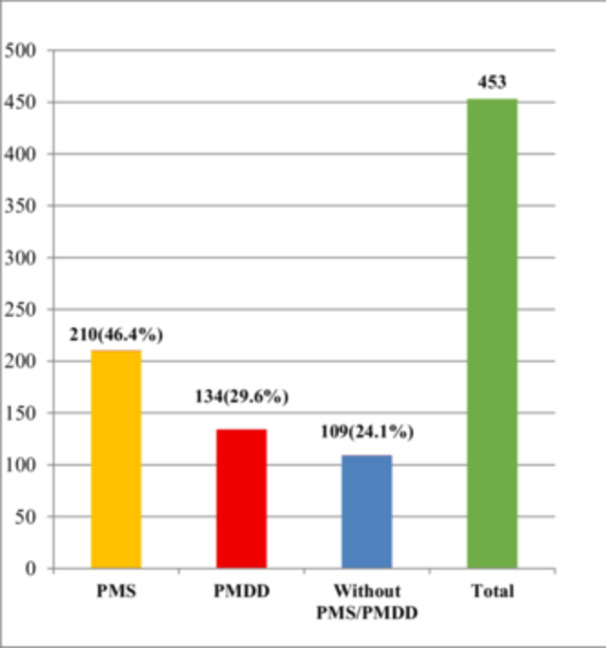
Prevalence of PMS and PMDD based on PSST criteria. PMDD is shown as present/absent only.

A total of 453 female students participated in the study. Overall, 75.9% (344/453) met the PSST diagnostic criteria for a premenstrual disorder (PMD). Of these, 210 (46.3%) were classified as PMS and 134 (29.6%) met criteria for PMDD (Figure 1). Among participants with PMS, 36.2% were mild, 52.8% moderate, and 11.0% severe based on PSST criteria.

### 3.3 Predictors of Premenstrual Disorders Among Female University Students in Western Uganda

We employed both bivariate and multivariate analyses to explore factors associated with PMDs. Variables with a *p*‐value < 0.2 in the bivariate analysis including age, religion, tribe, faculty, year of study, marital status, parity, ease of accessing school fees, regular exercise, menstrual history (age at menarche, knowledge of flow patterns, volume changes), healthcare‐seeking behavior, presence of menstrual conditions, drug use, BMI category, adverse menstrual symptoms, family history of mental illness, and history of traumatic events (Tables 2 and 3) were included in the multivariable logistic regression model.

**Table 2:** Bivariate analysis of sociodemographic and psychological factors associated with PMD.

Variable	PMD			
	No, *N* = 109, *n* (row %)	Yes, *N* = 344, *n* (row %)	cOR (95% CI)	*p* value
Age category (years)				
< 20	5 (35.7)	9 (64.3)	2.03 (0.48–8.63)	0.340
20–29	95 (22.5)	327 (77.5)	3.87 (1.45–10.31)	0.007
30–39	9 (52.9)	8 (47.1)	Ref	
Religion				
Christian	100 (26.2)	282 (73.8)	Ref	
Muslim	9 (12.7)	62 (87.3)	2.44 (1.17–5.10)	0.017
Nationality				
Ugandan	107 (24.4)	332 (75.6)	Ref	
Non‐Ugandan	2 (14.3)	12 (85.7)	1.93 (0.43–8.78)	0.393
Tribe				
Munyankole	38 (21.6)	138 (78.4)	Ref	
Muganda	51 (28.3)	129 (71.7)	0.70 (0.43–1.13)	0.143
Others	20 (20.6)	77 (79.4)	1.06 (0.58–1.95)	0.851
Faculty				
FCMD	38 (22.9)	128 (77.1)	3.37 (1.03–11.05)	0.045
SAHS	24 (13.0)	160 (87.0)	6.67 (1.99–22.36)	0.002
Pharmacy	9 (28.1)	23 (71.9)	2.56 (0.65–10.05)	0.179
Education	29 (60.4)	19 (39.6)	0.66 (0.18–2.34)	0.514
Law	3 (27.3)	8 (72.7)	2.67 (0.47–15.25)	0.270
Engineering	6 (50.0)	6 (50.0)	Ref	
Year of study				
First	31 (43.1)	41 (56.9)	Ref	
Second	40 (21.3)	148 (78.7)	2.80 (1.56–5.01)	0.001
Third	30 (21.6)	109 (78.4)	2.75 (1.48–5.09)	0.001
Fourth	2 (7.4)	25 (92.6)	9.45 (2.08–42.05)	0.004
Fifth	6 (22.2)	21 (77.8)	2.65 (0.95–7.34)	0.062
Marital status				
Married	20 (52.6)	18 (47.4)	Ref	
Not Married	89 (21.5)	326 (78.5)	4.07 (2.06–8.02)	< 0.0001
Parity				
0	94 (22.1)	331 (77.9)	4.06 (1.87–8.84)	< 0.0001
≥ 1	15 (53.6)	13 (46.4)	Ref	
School fees access				
Easy	71 (30.7)	160 (69.3)	Ref	
Difficult	38 (17.1)	184 (82.9)	2.15 (1.37–3.36)	0.001
Regular exercise				
Yes	42 (30.0)	98 (70.0)	Ref	
No	67 (21.4)	246 (78.6)	1.57 (1.00–2.47)	0.049
Family mental illness history				
Yes	4 (6.7)	56 (93.3)	5.10 (1.81–14.42)	0.002
No	105 (26.7)	288 (73.3)	Ref	
Personal mental health history				
Yes	2 (13.3)	13 (86.7)	2.10 (0.47–9.46)	0.333
No	107 (24.4)	331 (75.6)	Ref	
Traumatic event				
Yes	14 (9.7)	130 (90.3)	4.12 (2.26–7.53)	< 0.0001
No	95 (30.7)	214 (69.3)	Ref	

Abbreviations: aOR, adjusted odds ratio; CI, confidence interval; cOR, crude odds ratio; PMD, premenstrual disorder; PMDD, premenstrual dysphoric disorder; PMS, premenstrual syndrome.

**Table 3:** Bivariate analysis of gynecologic and medical factors associated with PMD.

Variable	PMD			
	No, *N* = 109, *n* (row %)	Yes, *N* = 344, *n* (row %)	cOR (95% CI)	*p* value
Cycle length				
24–38	109 (23.9)	344 (76.1)		
> 38	1 (100.0)	0 (0.0)	1.00	
Know normal flow				
YES	52 (21.0)	196 (79.0)	1.45 (0.94–2.24)	0.091
NO	57 (27.8)	148 (72.2)	Ref	
Menstrual duration				
≤ 8 days	109 (24.1)	343 (75.9)	1.00	
> 8 days	0 (0.0)	1 (100.0)		
Menarche				
< 15 years	98 (25.5)	286 (74.5)	Ref	
15+ years	11 (15.9)	58 (84.1)	1.81 (0.91–3.58)	0.090
Volume flow changes seen				
Yes	59 (18.8)	255 (81.2)	2.43 (1.55–3.80)	< 0.0001
No	50 (36.0)	89 (64.0)	Ref	
Volume flow concern				
Yes	46 (16.4)	235 (83.6)	2.95 (1.90–4.60)	< 0.0001
No	63 (36.6)	109 (63.4)	Ref	
Heavy menstrual bleeding				
YES	14 (19.2)	59 (80.8)	1.41 (0.75–2.63)	0.288
NO	95 (25.0)	285 (75.0)	Ref	
Conditions affecting menses				
YES	39 (16.9)	192 (83.1)	2.27 (1.45–3.54)	< 0.0001
NO	70 (31.5)	152 (68.5)	Ref	
Has menstrual symptoms				
YES	67 (18.6)	293 (81.4)	3.60 (2.21–5.86)	< 0.0001
NO	42 (45.2)	51 (54.8)	Ref	
Sought medical care for menses				
YES	26 (16.2)	135 (83.8)	2.06 (1.26–3.37)	0.004
NO	83 (28.4)	209 (71.6)	Ref	
BMI category				
Underweight	6 (30.0)	14 (70.0)	0.73 (0.27–1.97)	0.540
Normal	78 (23.9)	248 (76.1)	Ref	
Overweight	24 (26.7)	66 (73.3)	0.86 (0.51–1.47)	0.593
Obese	1 (5.9)	16 (94.1)	5.03 (0.66–38.56)	0.120
Waist circumference				
Normal	93 (24.0)	294 (76.0)	Ref	
High	16 (24.2)	50 (75.8)	0.99 (0.54–1.82)	0.970
Blood pressure category				
Normal	92 (23.9)	293 (76.1)	Ref	
Elevated	9 (29.0)	22 (71.0)	0.77 (0.34–1.73)	0.522
Hypertension	8 (21.6)	29 (78.4)	1.14 (0.50–2.58)	0.756
Drug abuse				
Yes	12 (9.1)	120 (90.9)	4.33 (2.28–8.21)	< 0.0001
No	97 (30.2)	224 (69.8)	Ref	

*Note:* Cycle‐length categories reflect WHO reference range; sensitivity analysis excluding cycles outside 24–38 days did not change results.

Abbreviations: aOR, adjusted odds ratio; CI, confidence interval; cOR, Crude odds ratio; PMD, premenstrual disorder; PMDD, premenstrual dysphoric disorder; PMS, premenstrual syndrome.

The multivariate analysis identified four independent predictors of PMDs. Compared with Christians, Muslim participants were nearly three times more likely to experience PMDs compared to Christians (aOR = 2.79, *p* = 0.020). Compared with those in the other faculties, students in the School of Allied Health Sciences had significantly higher odds of reporting PMDs (aOR = 4.25, *p* = 0.05). Drug use was a strong predictor, with users having more than twice the odds of having PMDs than non‐users did (aOR = 2.44, *p* = 0.024). Similarly, students with a history of traumatic events were more than double the odds of experiencing PMDs (aOR = 2.24, *p* = 0.031). Although factors such as age, marital status, BMI, menstrual conditions, family history of mental illness, and healthcare‐seeking behavior showed significant associations in the bivariate analysis, they did not remain significant in the multivariate model (Table 4).

**Table 4:** Multivariate analysis of factors associated with PMD.

	Premenstrual disorder		Bivariate analysis		Multivariate analysis	
Variable	No, *N* = 109, *n* (row %)	Yes, *N* = 344, *n* (row %)	cOR (95% CI)	*p* value	aOR (95%CI)	*p* value
Age category (years)						
< 20	5 (35.7)	9 (64.3)	2.03 (0.48–8.63)	0.340	0.45 (0.05–4.03)	0.473
20–29	95 (22.5)	327 (77.5)	3.87 (1.45–10.31)	0.007	0.98 (0.19–5.04)	0.984
30–39	9 (52.9)	8 (47.1)	Ref			
Religion						
Christian	100 (26.2)	282 (73.8)	Ref			
Muslim	9 (12.7)	62 (87.3)	2.44 (1.17–5.10)	0.017	2.79 (1.18–6.62)	0.020*
Tribe						
Munyankole	38 (21.6)	138 (78.4)	Ref			
Muganda	51 (28.3)	129 (71.7)	0.70 (0.43–1.13)	0.143	0.69 (0.39–1.24)	0.214
Others	20 (20.6)	77 (79.4)	1.06 (0.58–1.95)	0.851	1.29 (0.62–2.71)	0.499
Faculty						
FCMD	38 (22.9)	128 (77.1)	3.37 (1.03–11.05)	0.045	1.69 (0.37–7.62)	0.495
SAHS	24 (13.0)	160 (87.0)	6.67 (1.99–22.36)	0.002	4.25 (1.00–18.14)	0.050*
Pharmacy	9 (28.1)	23 (71.9)	2.56 (0.65–10.05)	0.179	1.30 (0.24–7.04)	0.762
Education	29 (60.4)	19 (39.6)	0.66 (0.18–2.34)	0.514	0.61 (0.12–2.98)	0.539
Law	3 (27.3)	8 (72.7)	2.67 (0.47–15.25)	0.270	0.71 (0.08–6.08)	0.752
Engineering	6 (50.0)	6 (50.0)	Ref			
Year of study						
First	31 (43.1)	41 (56.9)	Ref			
Second	40 (21.3)	148 (78.7)	2.80 (1.56–5.01)	0.001	1.26 (0.57–2.80)	0.572
Third	30 (21.6)	109 (78.4)	2.75 (1.48–5.09)	0.001	1.16 (0.47–2.89)	0.745
Fourth	2 (7.4)	25 (92.6)	9.45 (2.08–42.05)	0.004	4.41 (0.79–24.52)	0.090
Fifth	6 (22.2)	21 (77.8)	2.65 (0.95–7.34)	0.062	1.13 (0.30–4.24)	0.858
Marital status						
Married	20 (52.6)	18 (47.4)	Ref			
Not Married	89 (21.5)	326 (78.5)	4.07 (2.06–8.02)	< 0.0001	1.62 (0.34–7.74)	0.548
Parity						
0	94 (22.1)	331 (77.9)	4.06 (1.87–8.84)	< 0.0001	0.74 (0.14–4.01)	0.724
≥ 1	15 (53.6)	13 (46.4)	Ref			
School fees access						
Easy	71 (30.7)	160 (69.3)	Ref			
Difficult	38 (17.1)	184 (82.9)	2.15 (1.37–3.36)	0.001	1.45 (0.81–2.58)	0.209
Regular exercise						
Yes	42 (30.0)	98 (70.0)	Ref			
No	67 (21.4)	246 (78.6)	1.57 (1.00–2.47)	0.049	1.52 (0.86–2.66)	0.147
Know normal flow						
Yes	52 (21.0)	196 (79.0)	1.45 (0.94–2.24)	0.091	1.15 (0.63–2.11)	0.641
No	57 (27.8)	148 (72.2)	Ref			
Menarche						
< 15 years	98 (25.5)	286 (74.5)	Ref			
15+ years	11 (15.9)	58 (84.1)	1.81 (0.91–3.58)	0.090	1.87 (0.82–4.28)	0.139
Volume flow changes seen						
Yes	59 (18.8)	255 (81.2)	2.43 (1.55–3.80)	< 0.0001	0.94 (0.46–1.92)	0.866
No	50 (36.0)	89 (64.0)	Ref			
Volume flow concern						
Yes	46 (16.4)	235 (83.6)	2.95 (1.90–4.60)	< 0.0001	1.08 (0.53–2.20)	0.826
No	63 (36.6)	109 (63.4)	Ref			
Conditions affecting menses						
Yes	39 (16.9)	192 (83.1)	2.27 (1.45–3.54)	< 0.0001	1.03 (0.54–1.95)	0.925
No	70 (31.5)	152 (68.5)	Ref			
Has menstrual symptoms						
Yes	67 (18.6)	293 (81.4)	3.60 (2.21–5.86)	< 0.0001	1.41 (0.65–3.02)	0.385
No	42 (45.2)	51 (54.8)	Ref			
Sought medical care for menses						
Yes	26 (16.2)	135 (83.8)	2.06 (1.26–3.37)	0.004	1.23 (0.66–2.28)	0.521
No	83 (28.4)	209 (71.6)	Ref			
BMI category						
Underweight	6 (30.0)	14 (70.0)	0.73 (0.27–1.97)	0.540	0.67 (0.20–2.22)	0.514
Normal	78 (23.9)	248 (76.1)	Ref			
Overweight	24 (26.7)	66 (73.3)	0.86 (0.51–1.47)	0.593	1.10 (0.56–2.16)	0.786
Obese	1 (5.9)	16 (94.1)	5.03 (0.66–38.56)	0.120	4.55 (0.52–39.91)	0.171
Drug abuse						
Yes	12 (9.1)	120 (90.9)	4.33 (2.28–8.21)	< 0.0001	2.44 (1.13–5.30)	0.024*
No	97 (30.2)	224 (69.8)	Ref			
Family mental illness history						
Yes	4 (6.7)	56 (93.3)	5.10 (1.81–14.42)	0.002	2.95 (0.94–9.26)	0.064
No	105 (26.7)	288 (73.3)	Ref			
Traumatic event						
Yes	14 (9.7)	130 (90.3)	4.12 (2.26–7.53)	< 0.0001	2.24 (1.07–4.68)	0.031*
No	95 (30.7)	214 (69.3)	Ref			

Abbreviations: aOR, adjusted odds ratio; CI, confidence interval; cOR, Crude odds ratio; PMD, premenstrual disorder; PMDD, premenstrual dysphoric disorder; PMS, premenstrual syndrome.

* *p* ≤ 0.05.

## 4 Discussion

Overall, 75.9% of participants met the PSST screening criteria for PMS or PMDD, indicating a burden that extends beyond isolated symptoms. Compared with pooled global estimates for PMS (30%–40%) and PMDD (3%–8%) [1, 6], our prevalence is higher likely influenced, in part, by use of a retrospective screening instrument, which can capture more cases than prospective daily symptom charting. Even allowing for methodological effects, these data point to a substantial, clinically relevant burden within a Ugandan university setting, where academic demands and access to tailored mental‐health support may shape symptom expression and help‐seeking.

Our results align with reports from other low‐ and middle‐income contexts. Studies from Pakistan and Nigeria document considerable student burdens, PMS around 51% and notable PMDD rates [7, 19], with additional Nigerian and Ethiopian cohorts reporting high symptom loads among university women [20, 21]. Egyptian data likewise show elevated PMDD prevalence [22]. These parallels likely reflect shared stressors (academic workload, financial strain, limited campus services) and the widespread use of standardized tools such as the PSST. Some cohorts report even higher PMDD levels, for example, 66.9% and 64.6% in Ethiopian samples approaching graduation [23, 24], plausibly tied to end‐of‐program stress. Cross‐country comparisons further show wide variability: pooled or national figures range from lower estimates in parts of Europe and China to higher rates in Iran and across parts of the Middle East and North Africa [1, 6, 25]. Such differences likely arise from a combination of measurement approach (prospective vs. retrospective), thresholds, cultural norms around symptom disclosure, and contextual stress exposures rather than geography alone. Within Uganda, where student surveys report very high rates of menstrual complaints [5], our findings strengthen the case for locally adapted screening and support.

Severity patterns were notable for PMS. Among those screening positive for PMS, 36.2% were mild, 52.8% moderate, and 11.0% severe by PSST thresholds. In accordance with PSST conventions, PMDD is reported as a single category without severity grading and is therefore not subdivided here. These PMS distributions are comparable to student and young‐adult samples elsewhere for example, severe PMDD around 21.7% in Korea [26], single‐digit severe PMDD in some medical‐student cohorts [27], and roughly balanced mild–moderate versus severe PMS splits in Turkey [28] while Scandinavian data show lower severe PMD proportions [29]. Lower severity in some Western European settings (e.g., Spain: moderate–severe PMS 8.9%, PMDD 1.1%) [30] Contrasts with higher severity in resource‐limited or conservative contexts [31], and low PMDD incidence in parts of China [32]. For Ugandan students, concentrated exam schedules, limited structured counseling, and variable stigma may contribute to a heavier tail of moderate–severe symptomatology.

In multivariable analysis, four independent correlates remained: religion, academic faculty, drug use, and traumatic events. Muslim students had higher odds of PMDs than Christian peers (aOR 2.79, *p* = 0.020), consistent with prior reports [33, 34]; we interpret this as a contextual signal potentially indexing differences in coping norms, schedules, or help‐seeking rather than an intrinsic characteristic. Students in the School of Allied Health Sciences also showed higher odds, aligning with evidence that health‐professions training entails heavier workload, irregular hours, and exposure to illness and suffering, which may amplify PMD symptoms [35]. Drug use was associated with more than double the odds of PMDs (aOR 2.44, *p* = 0.024), consistent with disruptions of neuroendocrine pathways relevant to mood regulation [36]. A history of traumatic events (aOR 2.24, *p* = 0.031) likewise tracked with PMDs, in keeping with links between adversity, stress‐reactivity, and hypothalamic–pituitary–adrenal dysregulation [37]. Variables such as age, BMI, family history of mental illness, and prior help‐seeking were significant in bivariate analyses but attenuated after adjustment, suggesting that psychosocial and behavioral domains may be more proximate drivers of PMD expression in this student population.

### 4.1 Study Strengths and Limitations

The large cohort improved precision for PMS/PMDD estimates, and the PSST screening tool enabled graded severity profiling. Standardized procedures and a prespecified, parsimonious multivariable model supported robust identification of independent correlates while limiting overfitting. The cross‐sectional design and retrospective symptom recall may inflate prevalence relative to prospective daily ratings; recall and salience bias are possible. Imbalanced category sizes for some predictors produced sparse cells and wider Confidence Intervals, introducing small‐sample uncertainty despite Multicollinearity checks and effect‐size–focused interpretation. Future research should validate prevalence with prospective charting over ≥ 2 cycles, incorporate repeated‐measures designs to evaluate temporal patterns and impairment, and test campus‐level interventions (screening, stress‐management, trauma‐informed and substance‐use referral pathways) for uptake and impact.

## 5 Conclusions and Recommendations

Our study revealed a notably high prevalence of PMD among university students compared with global estimates. Moderate symptoms were the most frequently reported symptoms for both PMS and PMDD; however, PMDD was associated with a higher proportion of severe cases, indicating a greater symptom burden and the need for more focused clinical attention. The key factors associated with PMDs included being a student in the School of Allied Health Sciences, religious affiliation, drug use, and a history of traumatic experiences. We recommend that Kampala International University integrate mental health and reproductive health support services into the university system, with a particular focus on high‐risk groups. Early identification, awareness campaigns, and targeted interventions could play a critical role in reducing the impact of these disorders on students' well‐being and academic success. Future Ugandan studies pairing PSST screening with prospective daily ratings could refine prevalence estimates, clarify temporal dynamics, and test the impact of campus‐based supports on academic and psychosocial outcomes.

## Author Contributions

M.N., M.P.S.I., and T.H. designed and developed the proposal. M.N., A.O., C.K., S.A.S. and J.J.M. performed the data collection and entry. E.O. and T.H. performed the statistical analysis. R.M.I.N., A.K.K. drafted the initial manuscript. T.H. and E.O. contributed to reviewing and revising the manuscript. The final manuscript was read and approved by all the authors.

## Disclosure

The lead author Theoneste Hakizimana affirms that this manuscript is an honest, accurate, and transparent account of the study being reported; that no important aspects of the study have been omitted; and that any discrepancies from the study as planned (and, if relevant, registered) have been explained.

## Conflicts of Interest

The authors declare no conflicts of interest.

## Transparency Statement

Dr. Theoneste Hakizimana affirms this is an honest, accurate, and transparent account of the study; no important aspects have been omitted.

## Data Availability

The data supporting this study's findings are available from the corresponding author on reasonable request, subject to REC approval.
